# *Thelazia leesei* Railliet & Henry, 1910 (Spirurida: Thelaziidae) of dromedary camel *Camelus dromedarius*: further morphological description, molecular characterization, and epidemiology in Iran

**DOI:** 10.1186/s13071-024-06558-1

**Published:** 2024-11-24

**Authors:** Javad Khedri, Alireza Sazmand, Soheil Sadr, Mourad Ben Said, Shigehiko Uni, Domenico Otranto, Hassan Borji

**Affiliations:** 1 Department of Pathobiology, Faculty of Veterinary Medicine, Ferdowsi University of Mashhad, Mashhad, Iran; 2 Department of Pathobiology, Faculty of Veterinary Medicine, Bu-Ali Sina University, Hamedan, 6517658978 Iran; 3 Laboratory of Microbiology, National School of Veterinary Medicine of Sidi Thabet, University of Manouba, 2010 Manouba, Tunisia; 4 Department of Basic Sciences, Higher Institute of Biotechnology of Sidi Thabet, University of Manouba, 2010 Manouba, Tunisia; 5 Department of Health, Sports, and Nutrition, Faculty of Health and Welfare Studies, Kobe Women’s University, Kobe, 650-0046 Japan; 6 Department of Veterinary Medicine, University of Bari, 70010 Bari, Italy; 7 Department of Veterinary Clinical Sciences, City University of Hong Kong, Kowloon Tong, Hong Kong, China

**Keywords:** Camelinae, SEM, Microscopy, Phylogeny, PCR, Eyeworm, Thelaziosis

## Abstract

**Background:**

In camels, thelaziosis is mainly caused by *Thelazia leesei* Railliet & Henry, 1910, a little-known eyeworm species. Given the paucity of scientific data, this study aimed to provide new insights into the morphology, molecular characterization, and phylogenetic relationship of *T. leesei* and its occurrence in camels from Iran, where animals suffer from the high burden of eyeworms.

**Methods:**

From December 2020 to November 2022, slaughtered camels (*n* = 400) of different sex and age groups were examined in Sistan-va-Baluchestan province in Southeast Iran’s local abattoirs. Adult eyeworms were fixed and stored for morphological identification by light and scanning electron microscopy (SEM). Polymerase chain reaction (PCR) products corresponding to the partial sequences of the mitochondrial cytochrome *c* oxidase subunit I (*cox*1) of eyeworms were Sanger sequenced and analyzed phylogenetically.

**Results:**

A total of 118 (29.5%) camels from all five counties examined were infected with eyeworms, with an abundance of 0.9 and a mean intensity of 3.2 (i.e., up to 18 worms from a single animal). The infection rate was higher in camels older than 4 years of age (*P* = 0.01901). Lachrymation was associated with infection in animals (*P* < 0.00001). The morphology of our specimens resembled that of *T*. *leesei*, with the exception of the position of the nerve ring and esophagus length. Genetic analysis showed that the *cox*1 partial sequences of our *T. leesei* specimens had genetic distances of 8.8% to 13.5% compared with other *Thelazia* species.

**Conclusions:**

On the basis of the morphometrics and morphological characteristics, we identified our specimens as *T*. *leesei*. In the phylogenetic tree, *T. leesei* herein isolated formed a monophyletic group together with its congeners, and *T*. *leesei* formed a sister clade to *T. lacrymalis*. In addition, we demonstrated the epidemiology of the infestation of *T*. *leesei* in camels in the endemic areas of southeastern Iran. The data presented are crucial for better understanding the pathogenic role of *T. leesei* and developing effective treatment strategies. In particular, studies on the intermediate host(s) of *T. leesei* in these regions will support effective control strategies for this parasitosis.

**Graphical Abstract:**

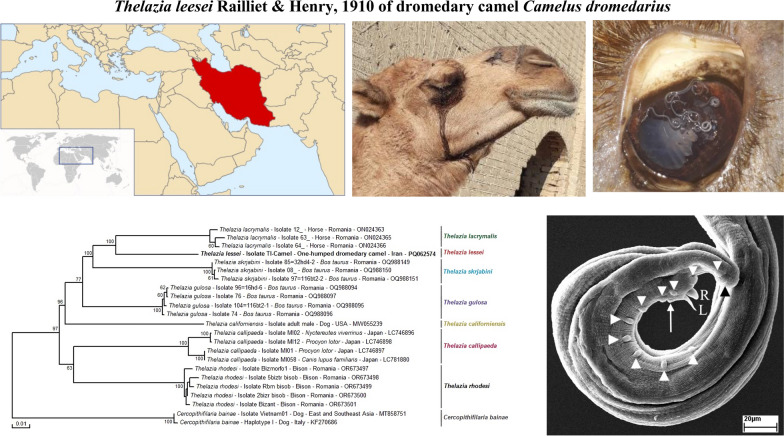

## Background

Species of the genus *Thelazia* Bosc, 1819 (Spirurida, Thelaziidae) are parasitic nematodes that reside under the lids, on the conjunctiva and nictitating membrane, in nasolachrymal ducts, conjunctival sacs, or excretory ducts of the lachrymal glands of a wide variety of mammals, including humans, and birds [1–3]. All *Thelazia* species are transmitted by zoophilic secretophagous, non-biting flies that feed on ocular secretions, tears, and conjunctiva of animals [4]. Flies ingest first-stage larvae of the eyeworms from the lacrimal secretions of an infected host and redeposit them as infective third-stage larvae, which eventually complete their life cycle by developing into adult eyeworms [4].

Human thelaziosis caused by *Thelazia callipaeda* Railliet & Henry, 1910 is frequently recorded in many areas of Southeast Asia, India, China, and Japan [1, 5]. Recently, thelaziosis has been reported in humans in some European countries [6, 7], and other species such as *Thelazia californiensis* Price, 1930 and *Thelazia gulosa* Railliet & Henry, 1910 were found in humans in North America [8–11]. These findings suggest that zoonotic cases caused by *Thelazia* species may occur where the infection is endemic in animal hosts. In camels, thelaziosis can be caused by *Thelazia leesei* Railliet & Henry, 1910, *Thelazia lacrymalis* (Gurlt, 1831), and *Thelazia rhodesi* (Desmarest, 1827) [12–17]. In particular, while *T*. *lacrymalis* infects mainly equines, especially horses in Europe, Asia, South America, and North America, and *T*. *rhodesi* infects bovids and less commonly horses [18], *T. leesei* is the only known parasite of Old-World camels (Camelini) [19]. *Thelazia leesei* was described in 1910 on the basis of the materials from the vitreous body of one-humped dromedary camel *Camelus dromedarius* in Lahore in Pakistan and Punjab in India [20, 21]. Further, this species was investigated in dromedaries in Kenya, Egypt, India, Iran, Turkmenistan, Azerbaijan, and Uzbekistan [13, 17, 20–27]. In addition, according to Railliet and Henry [21], Goubaux (possibly Armand Charles Goubaux) found this species in the left lacrimal gland of a dromedary in France in 1853. Despite the scarcity of information on its intermediate host, according to Dobrynin [15], *T*. *leesei* is transmitted by *Musca lucidula* (Loew 1856) in Turkmenistan. While *T*. *leesei* seems well tolerated by camels, clinical cases have been described in which it gained access to the vitreous chamber of the eye, inducing severe inflammation [21]. Morphological information about *T. leesei* is based on very limited descriptions due to a few specimens [20, 21, 24, 25], whereas no molecular data are available. Therefore, this study has been designed to provide new insights into the morphology of *T. leesei* by light and scanning electron microscopy (SEM), as well as the molecular characteristics and phylogenetic relationships within the genus *Thelazia*. In addition, herein we assessed the epidemiological risk factors associated with camel thelaziosis in a camel-rich region of Iran.

## Methods

### Study area and sampling

**Fig. 1:**
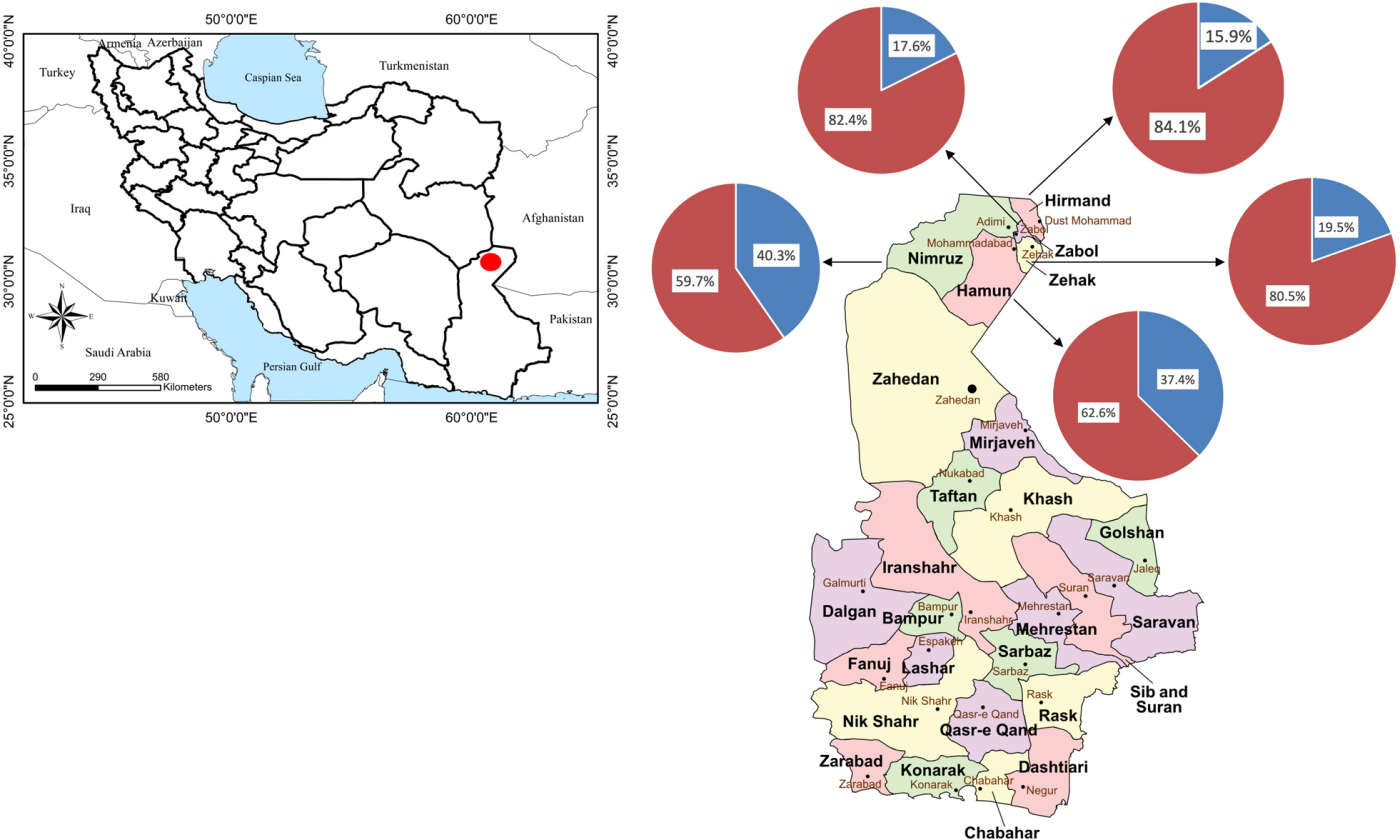
Map of Iran showing the sampling area with the distribution of positive (blue) and negative (red) samples in each county

This cross-sectional study was conducted in five counties of Sistan-va-Baluchestan Province in southeastern Iran (28°17′0″N, 61°7′0″E) named Zabol, Zehak, Hirmand, Hamoon, and Nimrooz (Fig. 1). The study area lies 475–500 m above sea level, and borders Afghanistan in the east and a desert in the west and northwest. It has a desert climate (Köppen-Geiger classification: BWh), with an annual precipitation of 59 mL and an average humidity of 40% [28].

From December 2020 to November 2022, a total of 400 slaughtered camels of different sexes and age groups (G1: < 2 years, G2: 2–4 years, G3: > 4 years) were examined in local abattoirs. Before slaughter, camels were reviewed for lachrymation and attraction of flies to the head as possible indicators of thelaziosis. The eyeball and surrounding tissues containing the lacrimal glands and upper and lower eyelids were removed. The conjunctival sacs and corneal surfaces of the eyes were examined by manipulating the orbital membranes to check for eyeworms’ presence. Then, the lateral canthus was cut, and the eye everted. Pressure was applied at the base of the lacrimal ducts to expel eyeworms from these sites, followed by the incision and examination of the nictitating membrane and the lacrimal ducts. Excised eyes and surrounding tissues from study camels were flushed with physiological saline solution (NaCl 0.9%), and the sediment was examined.

### Morphological analysis and scanning electron microscopy

Adult eyeworms isolated from the eyes were fixed and stored in glycerine-alcohol (5% glycerine in 70% ethanol). A total of 10 male and 10 female eyeworms were mounted on glass slides and identified morphologically within 1 week under a light microscope using valid keys [20, 25, 29, 30]. We compared the morphometrics of the present specimens with the original description of *T*. *leesei* [20, 21] and related records of other authors [24, 25, 31]. As *T*. *lacrymalis* and *T*. *rhodesi* were found in camels [12, 16], we compared the morphometrics of our specimens with records of the two species [5, 32, 33].

For SEM examination, 10 adult specimens (5 males and 5 females) were fixed in glutaraldehyde and osmium tetroxide, dehydrated in a graded series of ethanol, and mounted on stubs. The specimens underwent sputter coating with gold (Agar Scientific Ltd., Essex, UK) in an SC7620 fine coater (Quorum Technologies, East Sussex, UK). The micrographs were captured using a LEO1450VP scanning electron microscope (Carl Zeiss AG, Oberkochen, Germany) at 20 kV.

### Molecular analysis and phylogenic analysis

Genomic DNA was extracted from adult specimens using a commercial DNA extraction kit (MBST, Tehran, Iran) according to the manufacturer’s instructions. Primers targeting a 648 bp fragment of mitochondrial cytochrome *c* oxidase subunit I (*cox*1) (COlintF 5′-TGATTGGTGGTTTTGGTAA-3′/COIintR 5′-ATAAGTACGAGTATCAATATC-3′) were employed in conventional PCRs [34]. The PCR product (648 bp in size) corresponding to the partial sequence of the *cox1* gene of our specimen was purified and sequenced in both directions, using the same primers as for the PCR amplifications. The reactions were performed using a conventional Big Dye Terminator cycle sequencing ready reaction kit (Perkin Elmer, Applied Biosystems, Foster City, USA) and an ABI3730XL automated DNA sequencer.

The chromatograms were evaluated with Chromas Lite v2.01 (http://technelysium.com.au/wp/chromas/). Sequences were determined on both forward and reverse strands to obtain maximal data accuracy. Particularly, the complementary strands of each sequenced product were manually assembled using the DNAMAN software (Version 5.2.2; Lynnon Biosoft, Quebec, Canada). The primer region sequences were removed, and the overlapping parts were selected. Multiple sequence alignments and sequence similarities were calculated using the CLUSTAL W method [35]. By using the DNAMAN software, genetic distances among the operational taxonomic units were computed using the maximum composite likelihood method [36] and were used to construct the phylogenetic tree. Statistical support for internal branches of the trees was evaluated by bootstrapping more than 1000 replicates [36]. Two *Cercopithifilaria bainae* Almeida and Vicente 1984 *cox*1 partial sequences were added as an out-group. BLAST analysis of GenBank/EMBL/DDBJ was used to assess the level of similarity with previously reported sequences (http://blast.ncbi.nlm.nih.gov/) [37].

### Statistical analysis

The association of independent variables (i.e., intensity and abundance of infection, sex, age, lachrymation, and season) and infection was evaluated using the chi-squared and Fischer’s exact tests of SPSS software version 16 (Chicago, IL, USA). Descriptive statistics with 95% confidence intervals were used to analyze data, and the results were considered significant at *P* < 0.05.

## Results

### Infection of animals and risk factors

**Fig. 2:**
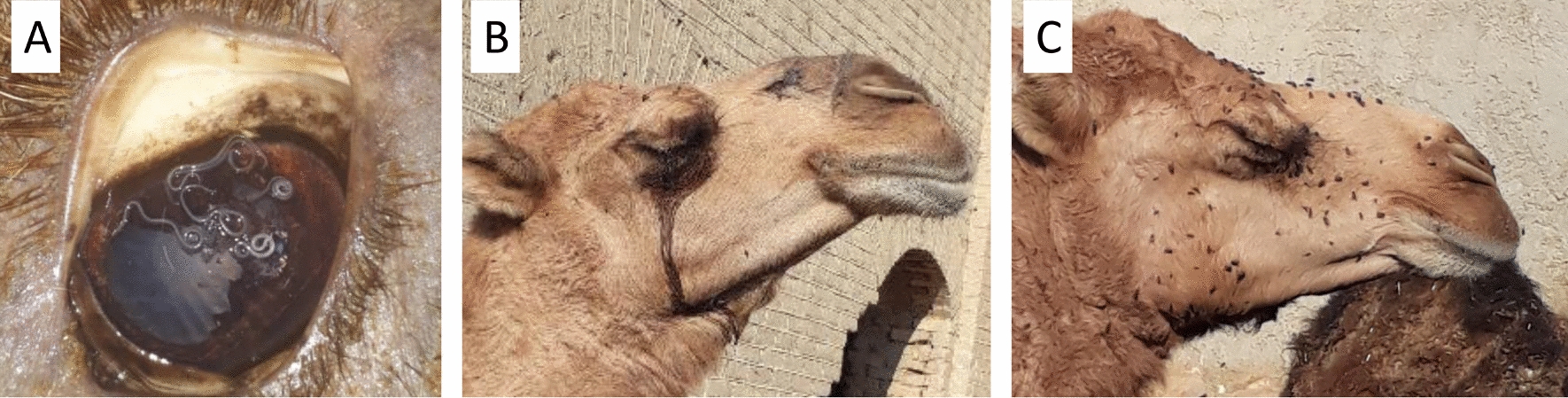
**A** Massive infection by *Thelazia leesei*, **B** Lachrymation was observed in infested camels, and **C** Abundance of flies around the head of camels

**Fig. 3:**
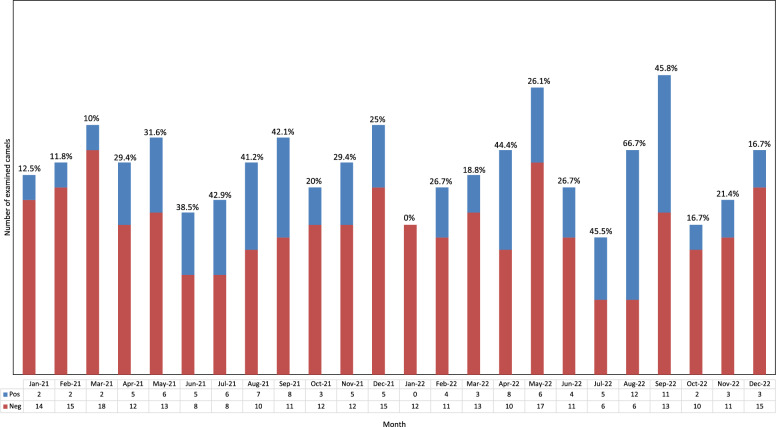
Prevalence of *Thelazia leesei* infection in camels of Sistan, as categorized by month

Out of 400 camels examined, 118 (29.5%) were infected with *T*. *leesei* eyeworms (Fig. 2A) with an abundance of 0.94 and a mean intensity of 3.19, with a maximum of 18 adult eyeworms collected from 1 camel in June. Thelaziosis was recorded in camels of all five counties examined (15.9–40.3%) but was significantly higher in Nimrooz county than the other four counties (*P* = 0. 000293) (Fig. 1). Camels of all three age groups harbored eyeworms in their eyes, but the infection rate was higher in camels G3 older than 4 years than the other groups (G1 and G2) (*P* = 0.01901). Both male and female camels were found infected with eyeworms, but there was no significant difference in infection rate between the two groups (*P* = 0.720781). Lachrymation was found to be higher in infested camels with flies around the face than without flies (*P* < 0.00001) (Fig. 2B, 2C). Thelaziosis was observed in all 12 months, having statistically higher positive cases in June than in the other months (*P* = 0.000239) (Fig. 3).

### Redescription of *Thelazia leesei* Railliet & Henry, 1910

#### Taxonomic summary

*Host*: *Camelus dromedarius* Linnaeus, 1758 (Cetartiodactyla: Camelidae), one-humped dromedary camel.

*Localities*: Zabol, Zehak, Hirmand, Hamoon, and Nimrooz, Sistan-va-Baluchestan Province, South-Eastern Iran.

*Voucher material deposited*: one female and one male in the Iranian National Parasitology Museum, accession no. 2022.N.52, code 1631.

*Anatomical location in host*: eyeball, lacrimal glands, eyelids, conjunctival sacs, and corneal surface of the eyes.

*Representative DNA sequences*: sequence data generated were deposited in the GenBank database. Sequence data for *T*. *leesei*: *cox*1 (PQ062574).

#### Optical microscopy of present specimens

**Fig. 4:**
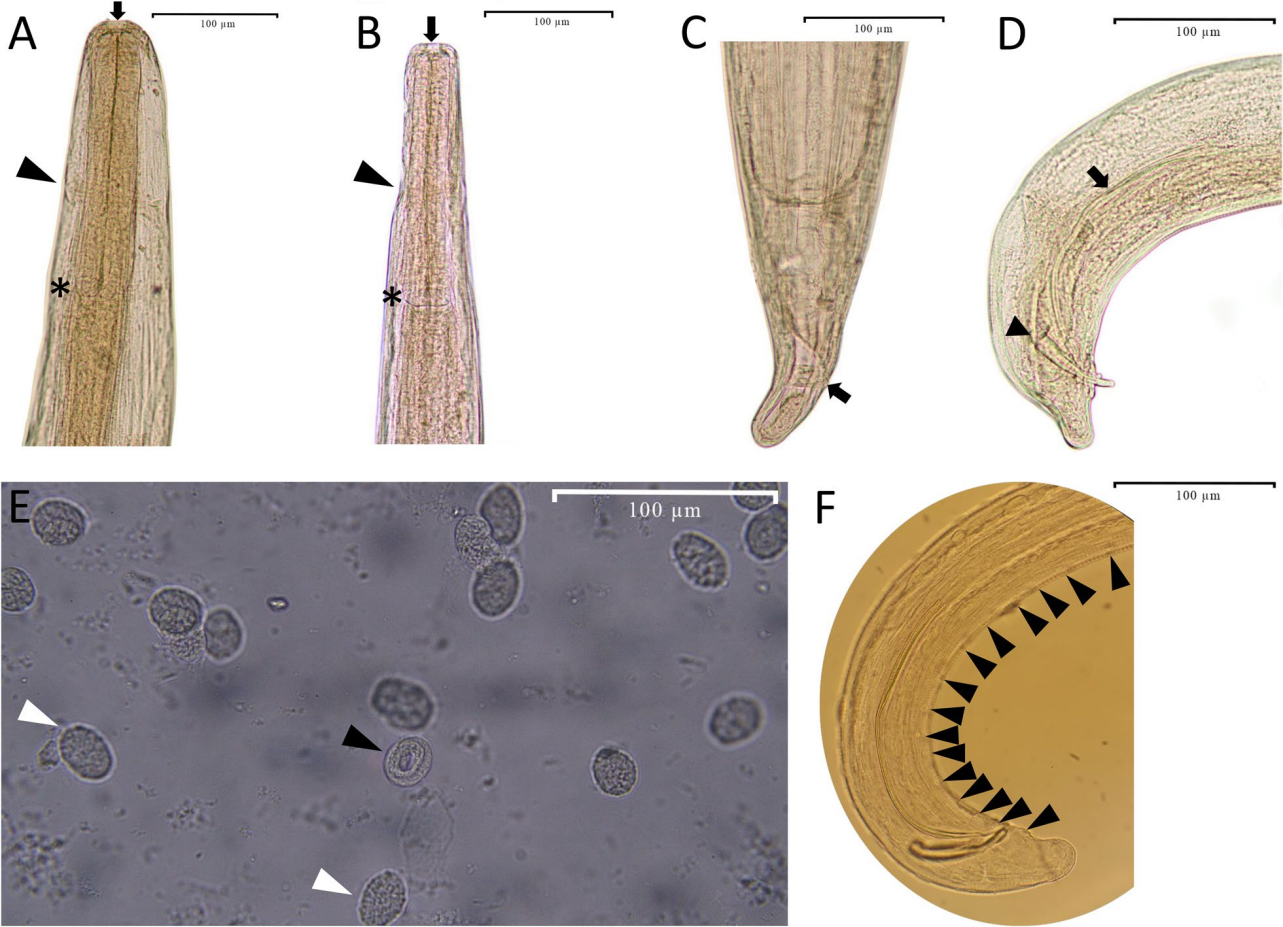
Light micrographs of *Thelazia leesei*. Females (**A** and **C**) and males (**B**, **D, F**). **A** and **B** Anterior region, showing buccal capsule (arrow) at the extremity and nerve ring (arrowhead), and esophago-intestinal junction (asterisk). **C** Posterior region, showing anus (arrow). Lateral view. **D** Posterior region, showing left spicule (arrow) and right spicule (arrowhead). Lateral view. **E** Unembryonated (white arrowhead) and larvated egg (black arrowhead) from the uterus. **F** Posterior region, showing precloacal caudal papillae (arrowheads). Lateral view

*General*: (Table 1, Fig. 4). Buccal capsule well developed resembling the shape of a vase (i.e., widening at the top and narrowing at the bottom). Esophagus not divided. Vulva situated in the anterior half of body. Posterior half of the body rounded in both sexes. Gubernaculum and caudal alae absent.

**Table 1:** Comparative morphometric data of *Thelazia leesi* from camels and congeners recorded from horses and cattle

Species	*Thelazia leesei*							*Thelazia lacrymalis*^a^		*Thelazia rhodesi*^a^		
Reference	Present study	Railliet and Henry [20]	Railliet and Henry [21]	Leese [38] (after Railliet and Henry [21])	Badanine, [25]	Baylis [24] (after Badanine [25])	Skrjabin et al. [31] (after Badanine [39])	Railliet and Henry [20]	Cotuțiu et al. [33]	Railliet and Henry [20]	Cotuțiu et al. [32]	Djungu et al. [40]
Host	Dromedary camels (*Camelus dromedarius*)	Dromedary camels (*Camelus dromedarius*)	Dromedary camels (*Camelus dromedarius*)	Dromedary camels (*Camelus dromedarius*)	Bactrian camels (*Camelus bactrianus*)	Bactrian camels (*Camelus bactrianus*)	Dromedary camels (*Camelus dromedarius*) and Bactrian camels (*Camelus bactrianus*)	Horses (*Equus caballus*)	Horses (*Equus caballus*)	Cattle (*Bos taurus*) and buffalo (*Bubalus bubalis*)	Domestic bovines (*Bos taurus*, *Bubalus bubalis*)	Cattle (*Bos taurus*)
Locality	Iran	Pakistan, India	Pakistan, India	Pakistan, India	Turkmenistan	Turkmenistan	Uzbekistan, Turkmenistan, Azerbaijan, India	–	Romania	France, Egypt, Indonesia	Romania	Indonesia
Female												
Body length (mm)	17–20.6 (average = 19.1, ± SD = 1.2)	17	15–21 (16.5)	10–21 (16.5)	11.4	15–21	14.4–15.4	14–18	7.5–17.2	12–18	–	12.5–20.5
Body width	455–499 (486, 14)	–	400	400	–	400	–	–	220–415	–	–	300–500
Buccal capsule, length	8.2–10.2 (9, 0.8)	–	–	–	–	12.5	13–14	–	12–20	–	–	–
Buccal capsule, width	15.2–17.6 (16.3, 0.8)	–	–	–	–	25	21–25	–	19–39	–	–	–
Nerve-ring from anterior end	134.5–139 (136.7, 1.5)	–	230–280	230–280	198	200–280^b^	200–210	–	202–322	–	–	–
Esophagus length	204.22–210.26 (207.5, 1.9)	320	335	335	360	320–360	360–390	–	309–411	–	–	–
Esophagus width at its largest portion	43.23–47.72 (45.5, 1.4)	–	60	60	–	–	–	–	61–85	–	–	–
Vulva from anterior end	423–443 (435.2, 6.5)	425	440	440	630	425–440	430–630	560	568–607	900–1000	1208	505.2–536.3
Tail length	68.5–70.4 (69, 0.6)	–	–	–	70	70	70–130	–	60–114	–	78	–
Unembryonated egg length	22.8–26.1 (24.7, 1.0)	–	–	–	35	–	28	–	13–28	–	–	–
Unembryonated width	14.1–18.9 (16.1, 1.7)	–	–	–	17.5–20	–	19	–	15.9–37.3	–	–	–
Larvated egg length	25.1–29.7 (26.9, 1.5)	–	–	–	–	–	–	–	–	–	–	–
Larvated egg width	14.3–20.9 (18.4, 1.6)	–	–	–	–	–	–	–	–	–	–	–
Larva length	45.7–55.8 (50.3, 3.9)	–	–	–	–	–	–	–	–	–	–	–
Larva width	5.4–9.2 (7.7, 1.1)	–	–	–	–	–	–	–	–	–	–	–
Male												
Body length (mm)	10.6–12.7 (11.7, 0.7)	–	12	6–12	12	12	10.1–11.9	8–12	6.5–9.6	8–12	–	7.5–14.5
Body width	241–279 (0.3, 0.01)	–	210	210	210	210	240–320	–	196–289	–	–	420–475
Buccal capsule, length	7–8 (7.3, 0.4)	–	–	–	–	–	9	–	10–17	–	–	–
Buccal capsule, width	8.2–10.3 (9.2, 0.7)	–	–	–	–	–	9–14	–	13–28	–	–	–
Nerve-ring from anterior end	106.6–110.1 (108.4, 1.2)	–	–	–	–	–	190–280	–	189–256	–	438	–
Esophagus length	190.2–196.4 (192.4, 1.7)	–	290	200	290	290	300–350	–	292–350	–	624	–
Esophagus width at its largest portion	36.1–38.6 (37, 0.9)	–	–	–	–	–	–	–	52–83	–	–	–
Left spicule length	340–360 (352.1, 6.2)	–	340	340	340	340	330–400	170–184	124–180	750–850	750	625–850
Right spicule length	91–120 (106.3, 9.7)	–	105	105	105	105	103	130–132	108–159	115–130	–	100–130
Ratio of left spicule to right spicule	3–3.7	–	3.2	3.2	3.2	3.2	3.2–3.9	1.3–1.4	1.1	6.5	–	6.3–6.5
Precloacal papillae	At least 30 papillae	–	25 papillae	At least 25 papillae	–	At least 25 [pairs of papillae]^c^	–	10 pairs	–	14	–	–
Tail length	40.7–42.3(41.7, 0.6)	–	–	–	–	–	60–70	–	74–160	–	–	–

Measurements are in micrometres unless otherwise stated

^a^*Thelazia lacrymalis* and *T*. *rhodesi* were recorded from camels [12, 16]

^b^Sex of the parasite was not indicated

^c^Doubtful

*Female*: [on the basis of 10 complete specimens]. Body length 17–21 (mean = 19.1, SD = 1.2) mm; width at mid-body 455–499 (486, 0.01) μm. Buccal capsule 8–10 (9.0, 0.8) μm long and 15–18 (16.3, 0.8) μm wide. Nerve ring 134–139 (136.7, 1.5) μm from anterior extremity (Fig. 4A). Esophagus 204–210 (206.4, 1.9) μm long and 43–48 (45.5, 1.4) μm wide. Vulva 423–443 (435.2, 6.5) μm from the anterior extremity. Tail 68–70 (69, 0.6) μm long (Fig. 4C).

Male: [on the basis of 10 complete specimens]. Body length 11–13 (11.7, 0.7) mm; width at mid-body 241–279 (260, 13) μm. Buccal capsule 7–8 (7.3, 0.4) μm long and 8–10 (9.2, 0.7) μm wide. Nerve ring 106–110 (108.4, 1.2) μm from anterior extremity (Fig. 4B). Esophagus 190–196 (192.4, 1.7) μm long and 36–39 (37.4, 0.9) μm wide. Precloacal papillae arranged in two ventrolateral groups with more than 15 pairs (Fig. 4F). Left spicule 340–360 (352.1, 6.2) μm long and right spicule 91–120 (106.3, 9.7) μm. Tail 41–42 (41.7, 0.6) μm long (Fig. 4D). Gubernaculum absent. Tail bent ventrally.

*Eggs and larvae*: [on the basis of 10 specimens]. No larvae inside the uteri. Unembryonated eggs in the uteri 22.8–26.1 (24.7, 1.0) μm long, 14.1–18.9 (16.1, 1.7) μm wide. Larvated eggs in the uteri 25.1–29.7 (26.9, 1.5) μm long, 14.3–20.9 (18.4, 1.6) μm wide. Grown larvae inside the eggs 45.7–55.8 (50.3, 3.9) μm long and 5.4–9.2 (7.7, 1.1) μm wide (Fig. 4E).

#### Scanning electron microscopy of present specimens

**Fig. 5:**
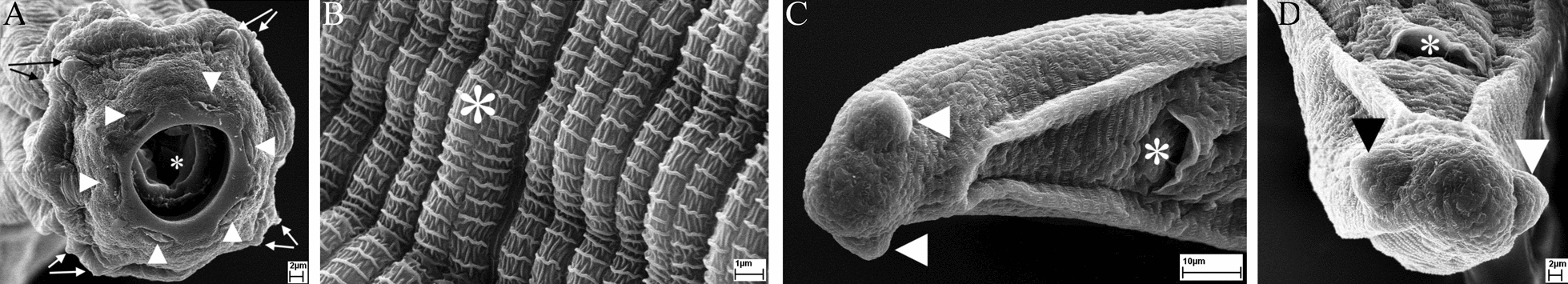
SEM images of *Thelazia leesei* females. **A** Apical view of the anterior region showing mouth opening (asterisk), six labial papillae (arrowheads), four twinned cephalic papillae (arrows), and two amphidial pores (§) at the lateral side. **B** Cuticular striations and longitudinal folds (asterisk) at mid-body region. **C** and **D** Posterior region showing anus (*) and large lateral papillae (arrowheads) at the tail extremity

Female: (Fig. 5). The oral end was provided with a round chitinous capsule, an inner circle of six sessile labial papillae, and an outer ring of four twinned cephalic papillae in addition to a pair of lateral amphids (Fig. 5A). The cuticle of the body was striated with longitudinal folds (Fig. 5B). The anus was situated ventrally at the posterior part (Fig. 5C). The tail bluntly rounded, bearing one pair of large lateral papillae near its extremity (Fig. 5D).

**Fig. 6:**
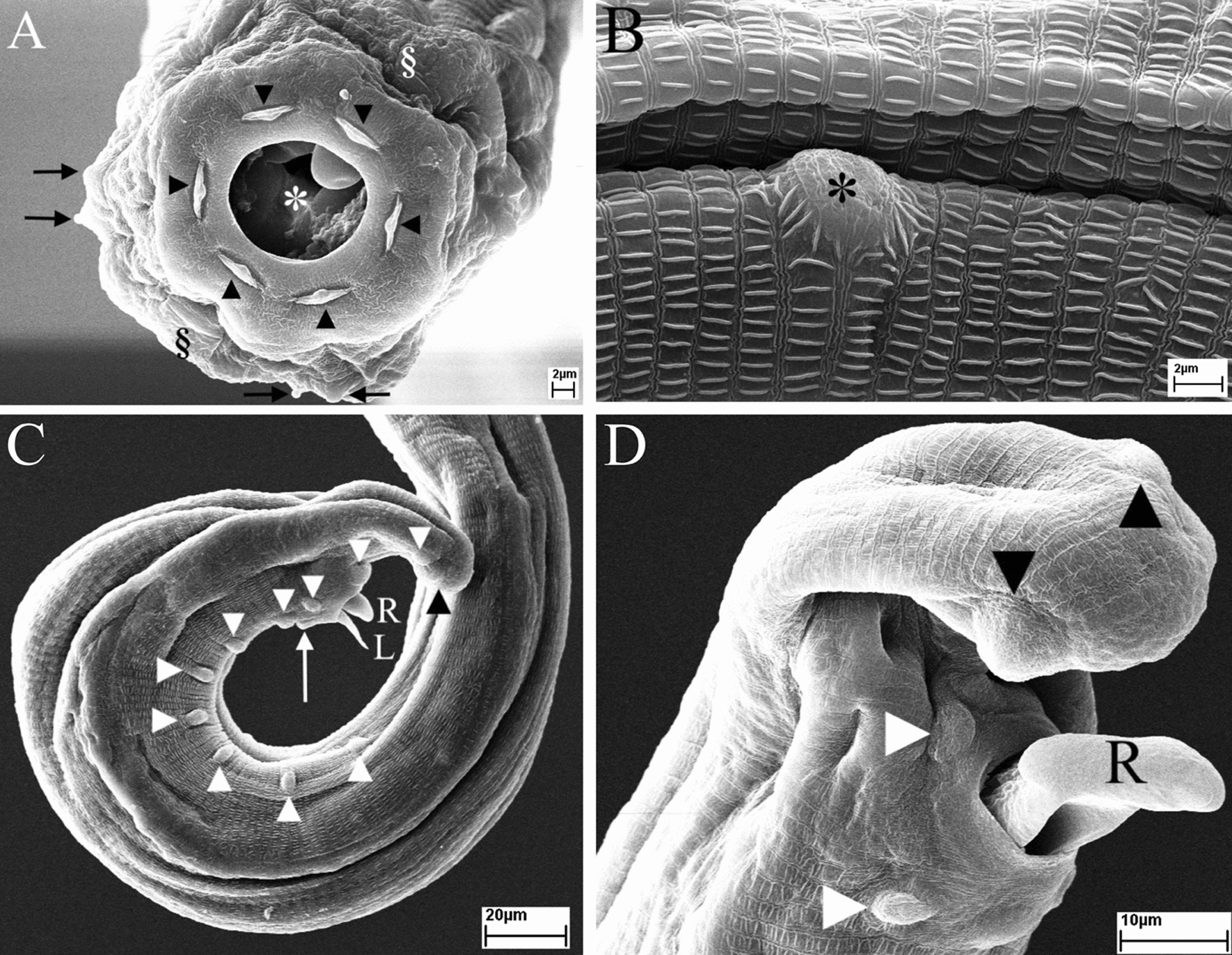
SEM images of *Thelazia leesei* males. **A** Apical view of the anterior region showing mouth opening (asterisk), six labial papillae (arrowheads), twinned cephalic papillae (arrows), and two amphidial pores (§) at the lateral side. **B** Posterior region showing caudal papilla (asterisk). **C** Posterior region showing caudal papillae (white arrowheads), precloacal central papilla (arrow), left spicule (L) and right spicule (R), and a large posterior papilla (black arrowhead). **D** Tail extremity showing single precloacal central papilla (arrow) in front of cloaca, right spicule (R) from cloaca, and large caudal papillae near the tail extremity (black arrowheads)

Male: (Fig. 6). The oral end (asterisk) was equipped with an inner circle of six sessile labial papillae (arrowheads) and an outer ring of four twinned cephalic papillae (arrows) in addition to a pair of amphidial pores (§) (Fig. 6A). Posterior part with caudal papilla (asterisk) and short longitudinal crests (Fig. 6B). Caudal papillae (arrowheads) formed two ventrolateral groups near cloacal aperture (Fig. 6C). One precloacal central papilla (arrow) located in front of the cloaca. The posterior part of the tail equipped with two pairs of postcloacal papillae (white arrowheads in Fig. 6C) and a pair of large lateral caudal papillae (black arrowheads) near the rounded tail extremity (Fig. 6D).

### Molecular identification and phylogenetic analysis

Uncorrected P-distances for the *cox*1 sequences between our specimen (PQ062574) of *T. leesei* and its congeners were 8.8% for *T. gulosa* (OQ988096), 10.6% for *T. lacrymalis* (ON024362), 11.2% for *T. rhodesi* (OR673500), 11.9% for *T. skrjabini* (OQ988148), 12.8% for *T. callipaeda* (MT040339), and 13.5% for *T. californiensis* (MW055240).

**Fig. 7:**
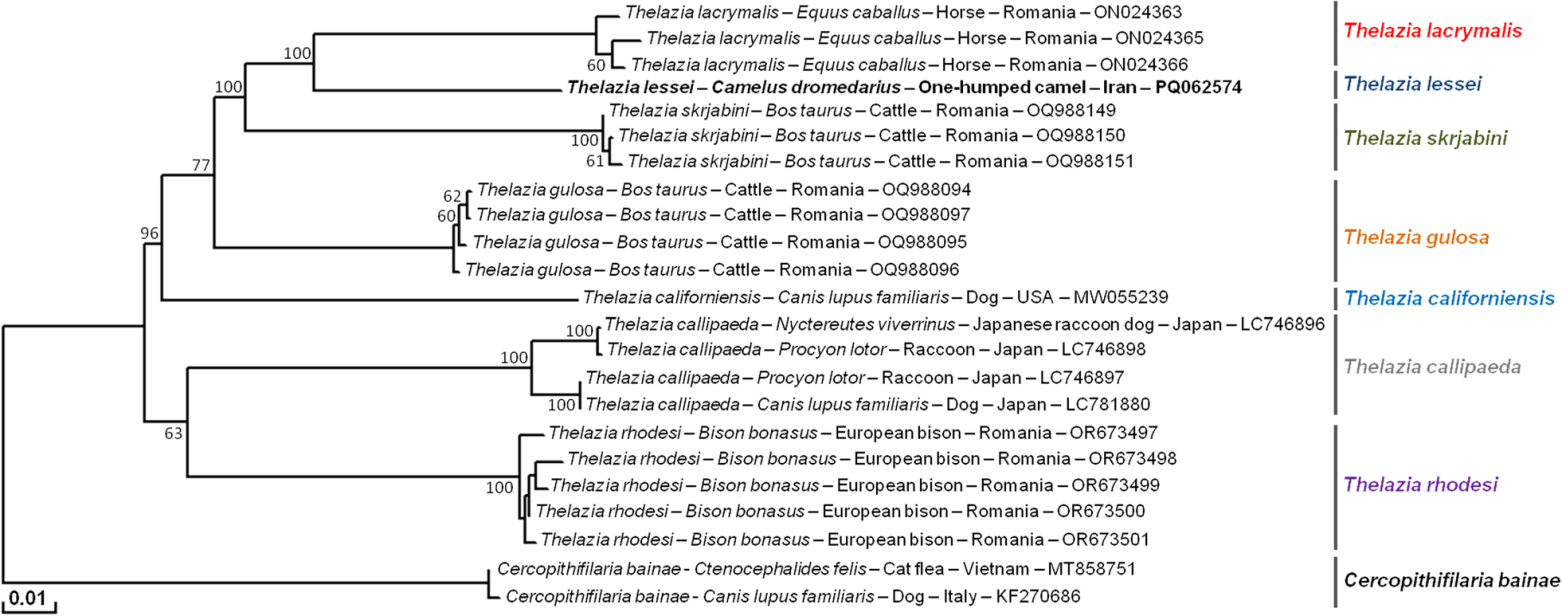
Phylogenetic tree of *Thelazia* species inferred with partial *cox*1 sequences (615 bp) of *Thelazia leesei* obtained in the present study. Numbers over the branches indicate the percentage of replicated trees in which the associated taxa clustered together in the bootstrap test (1000 replicates, only percentages greater than 50% were represented). The partial *cox*1 sequence representative of the *T*. *leesei* isolates obtained in this study is indicated in bold. The host, the country of origin, and the GenBank/EMBL/DDBJ accession number are indicated

In the phylogenetic tree, *T. lessei* formed a monophyletic group together with its congeners and indicated a sister clade to *T. lacrymalis* (ON024363, ON024365, and ON024366) from horse (*Equus caballus*) in Romania (Fig. 7).

## Discussion

The morphological characteristics and morphometrics of our specimens were similar to the original description of *T*. *leesei* by Railliet and Henry [20, 21] and records by others [24, 25].

As presented in Table 1, the distance of the nerve ring from the anterior end and the esophagus length of the present specimens were slightly different from those recorded in Skrjabin [31]. In addition, the position of the vulva and the tail length were slightly different from records by Badanine [25] and Skrjabin [31]. However, the other dimensions, such as body length, body width, and the buccal capsule were closely related to the records of *T*. *leesei* described previously [20, 21, 24, 25, 31, 38]. Conspicuously, the length of left and right spicules in our specimens was closely similar to the records of *T*. *leesei* [21, 24, 25, 31, 38]. In addition, our morphological features by SEM were closely related to the drawings, such as lateral papillae at the posterior end of the female in the records of *T*. *leesei* [25, 31]. While eggs in our study were smaller than those by Badanine [25] and Skrjabin [31], we regarded that the differences depend on the degree of development of eggs in the uteri. Comparing our specimens with *T*. *lacrymalis*, the length of the left spicule of our specimens was twice as long as that of *T*. *lacrymalis*. The ratio of left spicule to right spicule was three times as long as that of *T*. *lacrymalis*. On the contrary, the left spicule of our specimens was half length of *T*. *rhodesi* and the ratio of left spicule to right spicule was also half that of *T*. *rhodesi*. Hence, we considered that our specimens were *T*. *leesei* and closely related to *T*. *lacrymalis*, but different from *T*. *rhodesi* morphologically.

The phylogenetic analysis based on the *cox*1 sequences of *T. leesei* and the other six *Thelazia* species available in the GenBank/EMBL/DDBJ indicated a monophyletic group of the genus *Thelazia*. In *Onchocerca* species, *cox*1 interspecific distances are higher than 4.5% [41–43]. If this rule applies to *Thelazia* species, *T*. *leesei* is distinguished from other *Thelazia* species molecularly.

Herein, we added southeastern Iran to the regions endemic to *T. leesei*. Considering that the animals we examined came from a region bordering Pakistan and Afghanistan where camels freely roam [44], it can serve as an indication of the possible presence of *T. leesei* in these countries. This assumption could also be supported by the statement in Arnold Spencer Leese’s book published in 1927 titled *Treatise One Humped Camel in Health and in Disease*, in which he wrote “quite a large proportion of camels carry the parasite” [38]. Leese was a camel specialist working in present-day Pakistan, India, Kenya, and Somalia, thus it is unclear in which territory he observed this high prevalence. Indeed, this eyeworm was considered common in dromedaries in India, Kenya, Egypt, and Turkmenistan, but rare within the borders of Central Asia, Uzbekistan, and Turkmenistan [13–15, 25]. Since dromedaries are scattered in 47 countries in Africa and Asia and have a population in the Canary Islands of Spain [45], nationwide epidemiological studies would provide valuable insight into the distribution of *T. leesei*.

Very few studies have reported on the number of adult eyeworms present in the eyes of camels. Badanine found one female *T. leesei* in a dromedary in 1938 in Turkmenistan and two dromedaries in 1941 in Uzbekistan [25]. From two infected camels, 33 (25 in the right and 8 in the left eye) and 4 (2 in each eye) specimens were collected [25, 31]. Ivashkin (1961) examined three dromedaries in Uzbekistan, with two harboring four specimens (one male and three females); and four dromedaries in Turkmenistan, of which two had five specimens (two males and three females) [17]. In the only previous study from Iran, 3–10 eyeworms were collected per infected eye in dromedaries slaughtered in Tehran abattoir [23].

The infection rate in this endemic area of Iran peaked from July to September, although positive camels were diagnosed throughout the year. Similar findings were reported previously in Turkmenistan and Uzbekistan, with eyeworm infections observed in the second half of summer [25] and the infection had higher prevalence in autumn (49.6%) compared with winter (27.5%) [46]. This seasonal variation may be dependent on an increase in fly populations associated with higher temperatures and lower humidity [47].

There is only one study on the biology and life history of *T. leesei*, dating back to 1974, in which Dobrynin found that *Musca lucidula* (Loew, 1856) (Muscidae) is the competent vector of this eyeworm and that development to the third-stage larvae occurs takes 10–11 days at 21–32 °C [15]. As for *T. callipaeda* thus far, *Phortica variegata* (Fallén, 1823) (Drosophilidae), *P. okadai* (Máca, 1977), and *P. oldenbergi* Duda 1924 have been recognized as competent vectors in Europe and Asia [48, 49]; *M. autumnalis* De Geer, 1776, *M. larvipara* Portschinsky, 1910, *M. osiris*, Wiedemann 1830 and *M. domestica* for *T. gulosa*; and *M. autumnalis* and *M. larvipara* for *T. rhodesi* were identified as intermediate hosts in southern Italy [50]. Therefore, muscid flies other than *M. lucidula* may act as vectors of *T. leesei* in different camel-rearing regions of the world, advocating for further research on the topic. Significantly more camels older than 4 years were positive for *T. leesei*, possibly due to longer exposure to the vectors, as also demonstrated for other *Thelazia* spp. such as *T. callipaeda* [51, 52] and *T. rhodesi* [39].

Although we found lachrymal secretions and flies around the head of the *Thelazia*-infested camels before slaughter, these could be due to other factors, e.g., bacterial infections, physiological conditions, etc. A quantitative assessment of these factors is suggested. In particular, studies on the intermediate host(s) of *T. leesei* regions will support effective control strategies for this parasitosis.

## Conclusions

In this study, we redescribed the morphological features of *T. leesei* from the one-humped dromedary camel by light and scanning electron microscopy. On the basis of the morphometrics, *T*. *leesei* was closely related to *T*. *lacrymalis*, but differed distinctly from *T*. *rhodesi* morphologically. Molecular analyses indicated that species of *Thelazia* constitute a monophyletic group and *T*. *leesei* formed a sister clade to *T*. *lacrymalis*. We determined the prevalence of *T*. *leesei* infection in the camels in Southeast Iran and assessed the risk factors associated with the infestation of *T*. *leesei* in the host animals. The data presented are pivotal for better understanding the pathogenic role of *T. leesei* and implementing effective treatment strategies.

## Data Availability

The data presented in this study are contained within the article and supplementary material. Additional data can be provided on request.

## Abbreviations

PCR: Polymerase chain reaction
*cox*1: Cytochrome *c* oxidase subunit I
SEM: Scanning electron microscope/microscopy
μm: Micrometer
mm: Millimeter
NaCl: Sodium chloride
SD: Standard deviation
